# Neural architecture search via progressive partial connection with attention mechanism

**DOI:** 10.1038/s41598-024-57236-2

**Published:** 2024-03-18

**Authors:** Cong Jin, Jinjie Huang, Yuanjian Chen

**Affiliations:** 1 School of Computer Science and Technology, Harbin University of Science and Technology, Harbin, 150080 China; 2 School of Automation, Harbin University of Science and Technology, Harbin, 150080 China

**Keywords:** Neuroscience, Medical research, Preclinical research

## Abstract

Differentiable architecture search requires a larger computational consumption during architecture search, and there exists the depth gap problem under deeper network architecture. In this paper, we propose an attention-based progressive partially connected neural architecture search method (PPCAtt-NAS) to address these two issues. First, we introduce a progressive search strategy in the architecture search phase, build up the sophistication of the architecture gradually and perform path-level pruning in stages to bridge the depth gap. Second, we adopt a partial search scheme that performs channel-level partial sampling of the network architecture to further reduce the computational complexity of the architecture search. In addition, an attention mechanism is devised to improve the architecture search capability by enhancing the relevance between the feature channels. Finally, we conduct extensive comparison experiments with state-of-the-art methods on several public datasets, and our method is able to present higher architecture performance.

## Introduction

Neural Architecture Search (NAS)^1–5^ is intended to automatically search for the neural network architecture. It doesn’t require much priori knowledge for designing a good neural network architecture compared to traditional manually design methods^6–8^. However, NAS demands relatively much computing time and resources, for example, the early NAS methods took months to discover a suitable network architecture for a particular task or dataset^9–13^ and required hundreds or even thousands of computing devices. This fact makes the traditional NAS difficult for most scholars to conduct experimental studies.

To tackle these issues, Liu et al.^14^ proposed a more efficient architecture search approach known as Differentiable Architecture Search (DARTS), which constantly relaxed the discontinuous search space. This allows the architecture to be optimized by the gradient descent method. However, the huge search space still requires a large amount of computation during the architecture search phase. DARTS is mainly divided into two phases: the architecture search phase and the architecture evaluation phase. Due to the limitation of GPU memory size, DARTS has to start searching in a shallow architecture network at the first phase, and evaluating on the deeper architecture network at the second phase. This leads to another problem, that is, the depth gap between the architecture search phase and the architecture evaluation phase. Because the depth gap cannot guarantee the correlation between the two phases, the performance of the cells for deeper network architecture requirements is reduced in the architecture evaluation phase, and the extension of the method to other different datasets or tasks is also limited.

Many different search strategies have also been proposed to overcome problems of the depth gap and the huge computational complexity of DARTS. Xu et al.^15^ proposed a partial channel connectivity strategy to perform stochastic partial sampling of all intermediate nodes in the cell, further reducing the computational cost. Ding and Kim et al.^16–18^ proposed Broad Neural Architecture Search (BNAS) based on the Broad Convolutional Neural Network (BCNN) to speed up the architecture search process. Chen et al.^19^ proposed the idea of the progressive search strategy, which alleviates the adverse effects of the depth gap problem to some extent. Many different search strategies^20–23^ have also been proposed to alleviate the adverse effects of the depth gap problem. In addition, the attention mechanism can help the neural network select useful features and discard the less-useful ones. Attention mechanism modules have been introduced to enrich the search space^24,25^ to improve the architecture search performance. Some other methods have also used different search strategies^26–30^ to try to alleviate the above problems.

In this paper, firstly, we propose a new progressive search strategy that gradually increase the width and depth of the network architecture, to enable the architecture depth in the architecture search phase to gradually approach the depth required in the architecture evaluation phase. In the early stage of the search phase, we use a small number of searches to roughly search to the importance of the architecture parameters sorting, and in the later stage when requiring a more refined sorting, we then increase the number of searches. Secondly, to reduce the computational burden, we present a progressive partially connected search scheme, which gradually increases the channel sampling probability, making the distribution of image features in each stage of the search more rationalized. Thirdly, we introduce an attention mechanism to improve the network architecture search performance by adding an attention module to the cell, which makes the search process focus more on important features. Synthesizing the above three aspects, a novel progressive search method for partially connected network architecture with attention mechanism (PPCAtt-NAS) has been completed.

The main contributions are as follows:

1. We propose a new progressive network architecture search strategy that improves the efficiency of network architecture search and alleviates the adverse effects of the depth gap problem.
2. We adopt a progressive partial connection search scheme to improve the network architecture search performance and reduce the calculation in the architecture search stage.
3. We design a new attention module to the cells to achieve a more efficient architecture search process.
4. We perform extensive experiments on several publicly available datasets to verify that the optimal network architecture gained from our proposed search method exhibits higher architectural performance.

## Method

### Progressive architecture search strategy

**Figure 1:**
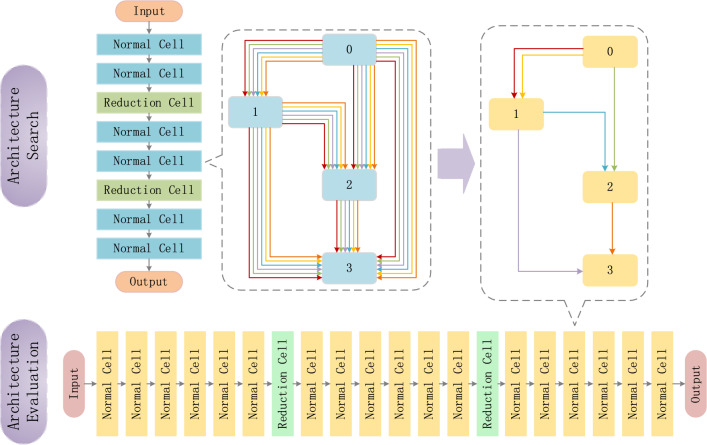
The overall architecture of DARTS.

We use DARTS as the baseline architecture, which represents the cell as a directed acyclic graph (DAG) containing $N$ nodes, each cell containing two input nodes, one output node and $N - 3$ intermediate nodes, according to the description in^14^. Each node in the graph represents the feature inputs for each layer, and each edge $E\left( {i,j} \right)$ represents a continuous weighting operation $o_{{\left( {i,j} \right)}}$ for transforming input $x_{i}$, denoted as

1 $$f_{i,j} \left( {x_{i} } \right) = \sum\limits_{o \in O} {\frac{{\exp \left( {\alpha_{o}^{{\left( {i,j} \right)}} } \right)}}{{\sum\nolimits_{{o^{\prime} \in O}} {\exp \left( {\alpha_{{o^{\prime}}}^{{\left( {i,j} \right)}} } \right)} }}} o\left( {x_{i} } \right)$$

where $o_{{\left( {i,j} \right)}}$ belongs to the operation space $O$. The operations in the search space consist of separable and dilated separable convolutions with convolution kernel sizes 3 and 5, max pooling and average pooling with convolution kernel size 3, skip connection and none. The overall architecture is shown in Fig. 1. The architecture in DARTS stacks 8 cells in the architecture search phase, but stacks 20 cells for the evaluation phase. Such a large span creates a depth gap between the two phases. This leads to the fact that the cell searched in the search phase does not play the same role expected in the architecture evaluation phase.

To compensate for the depth gap, we adopt a progressive network architecture search strategy, which gradually increases the sophistication of the architecture by steps, so that the network architecture at the end of the architecture search phase is closer to that of the architecture evaluation phase. The selection of operations in the search space during the architecture search phase is mainly based on the ranking of the importance of the operations, denoted by $\alpha$. A shallower and narrower network architecture with fewer searches is used to explore the architecture search space at the beginning of the architecture search thus obtaining a rough ranking of the $\alpha$. In the later stages, as the ranking of the $\alpha$ gradually settles down, the operations of similar importance cannot be further distinguished. We prune the search space $\mathrm{\rm O}=\left\{{o}_{1},{o}_{2},\dots ,{o}_{i},\dots \right\}$, gradually discard the useless operations to reduce the number of operations in the search space. Thus, we can use a deeper and wider network architecture with more searches in a smaller search space to obtain a more accurate ranking of the $\alpha$.

**Figure 2:**
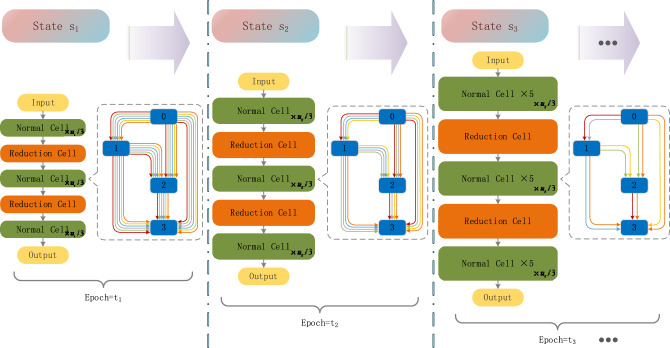
Progressive architecture search.

Concretely, we divide the architecture search phase into $S=\left\{{s}_{1},{s}_{2},\dots ,{s}_{i},\dots \right\}$ stages. Each stage ${s}_{i}$ consists of a stack of ${l}_{i}$ cells (including ${u}_{i}$ normal cells (N-Cells) and ${l}_{i}-{u}_{i}$ reduction cells (R-Cells)) with an initial number of channels ${c}_{i}$, and search ${t}_{i}$ times in a search space with a number of ${o}_{i}$ operations. The detailed architectural search flow chart is shown in Fig. 2. We increase the depth of the network architecture by gradually growing ${l}_{i}$, and increase the width of the network architecture by gradually growing ${c}_{i}$. At the end of each stage, only ${top{\_}k}_{i}$ operations are selected and ${d}_{i}$ operations with lower weights are discarded. For the discarded operations, their weights are not updated in the next stage. And we gradually increase the number of searches ${t}_{i}$ in each stage. In addition, we use dropout in each stage and gradually increase the dropout rate to build a more fairly competing with other operations in the architecture search space during the architecture search.

### Progressive partial connection search strategy

To reduce the cost of calculation, we propose a progressive partial connection search strategy. In the search space $\mathrm{\rm O}$ ($o\in \mathrm{\rm O}$), taking the edge from ${x}_{i}$ to ${x}_{j}$ as an example, channel $q$ of the input ${x}_{i}$ is partially sampled with sampling probability $p$ and then the sampled portion is used for operation selection. After the calculation, it is then connected in series with the remaining unsampled input as the output ${x}_{j}$, denoted as

2 $${x}_{j}=\left\{\begin{array}{c}\sum_{o\in \mathrm{\rm O}}\frac{exp\{{{\alpha }_{o}}^{\left(i,j\right)}\}}{\sum_{{o}^{\mathrm{^{\prime}}}\in \mathrm{\rm O}}exp\{{{\alpha }_{{o}^{\mathrm{^{\prime}}}}}^{\left(i,j\right)}\}}\cdot o({x}_{i})\\ {x}_{i}\end{array} \begin{array}{c}sampled\\ unsampled\end{array}\right.$$

We introduce ${\Gamma }_{i,j}$ to mark whether the channel is sampled or not, if the channel is sampled then ${\Gamma }_{i,j}$ is 1, otherwise 0. Then the computation between every two nodes can be further expressed as

3 $${\rho }_{i,j}\left({x}_{i};{\Gamma }_{i,j}\right)=\sum_{o\in \mathrm{\rm O}}\frac{{\text{exp}}\left\{{{\alpha }_{o}}^{\left(i,j\right)}\right\}}{\sum_{{o}^{\mathrm{^{\prime}}}\in \mathrm{\rm O}}{\text{exp}}\left\{{{\alpha }_{{o}^{\mathrm{^{\prime}}}}}^{\left(i,j\right)}\right\}}\cdot o\left({{\Gamma }_{i,j}*x}_{i}\right)+(1-{\Gamma }_{i,j})*{x}_{i}$$

**Figure 3:**
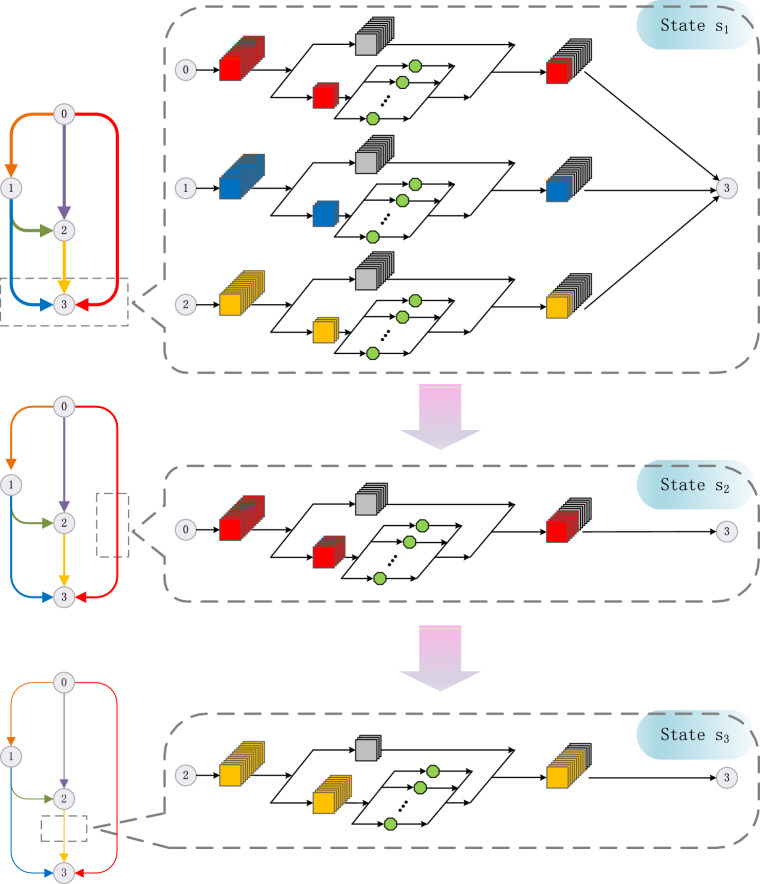
Progressive partial connection search strategy structure diagram.

We incrementally increase the channel sampling probability $p$ in the search phase in stages. In the early stage, the sampling probability is small, and a coarse operation importance ranking is obtained based on a few image features with a small number of channels. In the later stages, the sampling probability is gradually increased to select the best operation based on more image features with a larger number of channels. The structure diagram of the progressive partial connection search strategy is shown in Fig. 3.

### Attention module in the network architecture

**Figure 4:**
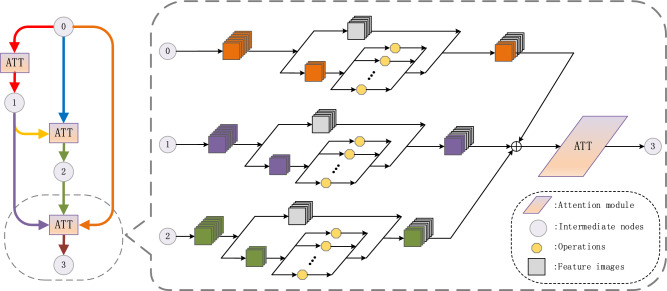
Attention network architecture.

To strengthen the relevance between feature channels within the cell, enable the search process focus more on the important features, and extract the feature information of interest. We introduce the attention mechanism in the architecture search phase, add a new attention module in the cell. Specifically, we first convert the input ${{x}_{i}}^{C\times H\times W}$ to ${{{x}_{i}}^{\mathrm{^{\prime}}}}^{C\times 1\times 1}$ by averaging pooling, where $C$ and $H\times W$ represent the channel count and dimensions of the input image, respectively. Then the converted image is fed into a multilayer perceptron containing three hidden layers, and finally multiplied by the input image, expressed as

4 $${y}_{i}=\delta \left(MLP\left({x}_{i}^{\mathrm{^{\prime}}}\right)\right)\cdot {x}_{i}=\delta ({\Omega }_{2}({\Omega }_{1}({\Omega }_{0}({x}_{i}^{\mathrm{^{\prime}}}))))\cdot {x}_{i}$$

where $\delta$ is the Sigmoid activation function, $\Omega$ is the weight of the MLP, ${\Omega }_{0}\in {R}^{(C/r)\times C}$, ${\Omega }_{1}\in {R}^{(C/r)\times (C/r)}$, ${\Omega }_{2}\in {R}^{C\times (C/r)}$, $r$ is the reduction ratio, and ${y}_{i}$ is the output of the attention module. After adding the above attention module to each intermediate node of the cell, it strengthens the interrelationship between each channel, which helps the architecture capture the spatial correlation between features, makes the architecture search pay more attention to the important operations in the search space, and selects a more suitable network architecture. It is proved that the attention module can greatly improve the architecture search performance. The structure of the network architecture basic unit after adding the attention module is shown in Fig. 4.

### Relationship to prior work

Although the P-DARTS^19^ and GPAS^28^ methods have similar thoughts of staged progressive search with PPCAtt-NAS method, PPCAtt-NAS method is more comprehensive. The P-DARTS method only takes into account the progressive approximation at the level of the number of operations, the number of cell stacks, and the dropout probability. The GPAS method considers gradual approximation at the level of the number of operations, the number of initial channels, the number of cell stacks, the number of epochs, and the proportion of the training set, and adds an artificial early stopping strategy, but we believe that we have to minimize the human intervention to achieve the purpose of automatic search. In contrast to them, this paper considers the whole aspect of the progressive idea of the number of operations, the number of initial channels, the number of cell stacks, the number of epochs, and the dropout probability at the same time. In terms of the dataset to guarantee a greater degree of feature capture, this paper does not use a progressive approach.

This paper further adds the idea of progressive partial channel connection, the sampling proportion of the channel is also added to the progressive idea, thereby increasing the diversity of feature selection with the depth of the search process. The PC-DARTS method^15^, the RS-DARTS method^26^, and the AutoRSISC method^27^ also use the idea of partial channel sampling but do not take into account the progressivity of the sampling. In addition, the attention module is added in this paper, while none of the above methods consider the importance of the attention mechanism within the cell.

## Experiments

### Implementation details

We conduct experiments on three datasets for image classification, namely Fashion-MNIST, CIFAR10, and CIFAR100. We perform architectural search on the CIFAR10 dataset and then evaluate the architecture on each of the three datasets. The architecture search space is the same as DARTS. Our experiments were conducted on an NVIDIA GeForce RTX 2080Ti GPU.

According to the PPCAtt-NAS search strategy, we divide the architecture search phase $S$ into three stages, where the first stage ${s}_{1}$, consisting of a stack of ${l}_{1}=5$ cells (which contains ${u}_{1}=3$ N-Cells) with an initial channel count of ${c}_{1}=8$, searches ${t}_{1}=20$ times in a search space with an operation count of ${o}_{1}=8$, and at the end of the first stage, selects ${top{\_}k}_{1}=5$ operations, discards ${d}_{1}=3$ operations with lower weights, and samples the input channels with sampling probability ${p}_{1}=0.25$. In the second stage ${s}_{2}$, we set ${l}_{2}=11$, ${u}_{2}=9$, ${c}_{2}=16$, ${t}_{2}=25$, ${o}_{2}=5$, ${top{\_}k}_{2}=3$, ${d}_{2}=2$, and ${p}_{2}=0.5$. In the third stage ${s}_{3}$, we set ${l}_{3}=17$, ${u}_{3}=15$, ${c}_{3}=24$, ${t}_{3}=30$, ${o}_{3}=3$, ${top{\_}k}_{3}=1$, ${d}_{3}=2$, and ${p}_{3}=0.75$. The batch size is 128. The dropout probabilities of the three stages are 0, 0.4, and 0.7, respectively. In each stage, the architectural parameters $\alpha$ are fixed in the first 10 epochs and only the network parameters $\omega$ (such as the weights of the convolution filter) are updated. Then $\alpha$ and $\omega$ are updated simultaneously in the remaining epochs. In the evaluation stage, the initial channel count is 36, the batch size is 128, and the epochs are 600. For other settings, please refer to^14^ and^19^.

### Architecture search

**Figure 5:**
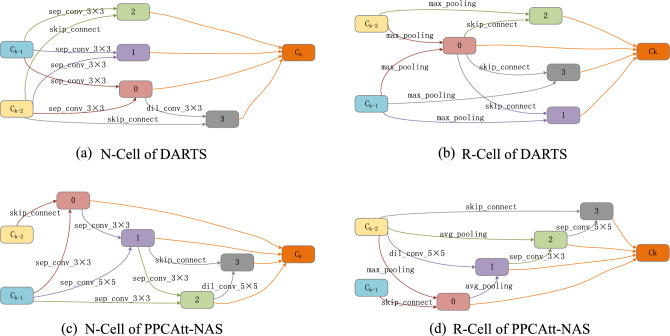
Best cells.

The best cell comparison of the two methods is shown in Fig. 5. It can be observed in Fig. 5 that the relationship between intermediate nodes of PPCAtt-NAS is more inclined to progressive relationship rather than juxtaposition in DARTS, indicating that our proposed search method focuses more on the interrelationship between intermediate nodes. Thus, the model architecture is deeper and more complex and can obtain better architectural performance. In addition, the R-Cell of PPCAtt-NAS selects many convolutional operations rather than non-parametric operations which the DARTS is more inclined to select, thus the sophistication of the network architecture is increased and the accuracy of the architecture is improved.

It is also noted that too many skip-connects may degrade the performance of the network architecture and thus cause performance collapse. Our method does not over-select skip-connects in the same or more epochs. Instead, the number of skip-connects is stabilized within 1 and 2. PPCAtt-NAS gradually selects non-parametric operations, avoiding the performance collapse that may occur during the search phase of the network architecture. This performance gain can be attributed to the introduction of the attention mechanism. The attention module added by PPCAtt-NAS to the network architecture increases the complexity of the network architecture and makes the opportunity of all operations to be selected in the search space more fairly, otherwise, the architecture search more inclined to select non-parametric operations when the network architecture is deeper.

We compare the experimental parameters of PPCAtt-NAS in the architecture search phase with those of DARTS and P-DARTS, and the results are shown in Table 1. From Table 1, compared with DARTS, the time consumed for searching is nearly reduced by a half. Although the total number of parameters increases, the architecture search performance of the PPCAtt-NAS is still stable after 75 searches in the search phase, whereas in DARTS, the performance collapse happened after only 50 searches. Compared with P-DARTS, our method is obviously superior. In addition, we can use a larger batch size to obtain more feature information.

**Table 1:** Comparison in architecture search stage.

Method	FP (MB)	SP (MB)	TP (MB)	TTP (MB)	TT (hours)	BS	Epoch
DARTS	1.93			1.93	0.37	64	50
P-DARTS	1.28	1.96	1.61	4.85	0.20	96	75
PPCAtt-NAS	0.09	0.93	3.52	4.54	0.18	128	75

FP, SP, TP, TTP, TT, BS, Epoch stand for first stage parameter number, second stage parameter number, third stage parameter number, total search parameter number, total search time, batch size number, and epoch number respectively.

To further validate the effectiveness of the method proposed in this paper, we conduct an ablation study to experimentally verify the strategies introduced in "Progressive architecture search strategy" to "Attention module in the network architecture" sections of this paper, respectively, and the results are shown in Table 2. We rerun the DARTS method that serves as the baseline and add the progressive architecture search strategy, progressive partial connection search strategy, and attention module proposed in this paper, respectively. Through Table 2 we can see that the three methods proposed in this paper all improve the architectural accuracy of the network architecture. Adding the attention module slightly increases 0.001 GPU-Days of search time and 0.07 M parameters, but improves the architectural accuracy by 0.19%, which is a significantly better improvement in terms of accuracy. In summary, it is verified that the search strategy proposed in this paper has feasibility.

**Table 2:** Experimental ablation studies.

Method	EP (M)	SC (GPU-days)	TE-A (%)	TE-B (%)
DARTS	2.84	0.365	2.94	2.83
DARTS + PAS	4.25	0.176	2.90	2.81
DARTS + PAS + PPCS	3.56	0.183	2.70	2.51
DARTS + PAS + PPCS + ATT (PPCAtt-NAS)	3.63	0.184	2.51	2.43

PAS, PPCS and ATT stand for the strategies proposed in "Progressive architecture search strategy" to "Attention module in the network architecture" sections of this paper, respectively. TE-A, TE-B, EP, SC, stand for average test error, best test error, evaluation stage parameters, and the time consumed in the search stage, respectively.

### Diagnostic experiments

In the architecture evaluation phase, we evaluate the network architectures on four datasets, Fashion-MNIST, CIFAR100, CIFAR10, and ImageNet, and compare them with other state-of-the-art manual-based methods and NAS methods. The comparison results on CIFAR10 and CIFAR100 are shown in Table 3. According to Table 3, the test error of PPCAtt-NAS is 2.51% (± 0.08), and the search phase time cost is 0.18 GPU days on CIFAR10. PPCAtt-NAS has a test error of 16.42% (± 0.24), and the architecture search phase time cost of 0.18 GPU days on CIFAR100. On CIFAR10, the search time cost of PPCAtt-NAS is significantly lower than that of most other methods. Although the architectural accuracy of some methods is slightly higher than that of PPCAtt-NAS, they require nearly twice the search time cost compared to our method. On CIFAR100, the architecture search cost of PPCAtt-NAS is also significantly lower than that of other methods, and the architecture search accuracy is comparable to that of most other search methods.

**Table 3:** Comparison Results on CIFAR10/CIFAR100.

Architecture	TE (%) (CIFAR10/CIFAR100)	EP (M)	SC (GPU-days)	SM
DenseNet-BC^7^	3.46/21.56	25.6/26.0	–	Mn
ResNet^8^	4.61/22.22	1.7/25.3	–	Mn
SENet^6^	4.05/21.42	11.2/26.5	–	Mn
NASNet-A^10^	2.65/18.34	3.3/3.3	2000	RL
AmoebaNet-A^12^	3.34 (± 0.06)/18.38	3.2/3.1	3150	Ev
PNAS^13^	3.41 (± 0.09)/19.53	3.2/3.2	225	SO
ENAS^2^	2.89/17.92	4.6/3.4	0.5	RL
DARTS^14^	2.76 (± 0.09)/17.76	3.3/3.3	1	Gd
PC-DARTS^15^	2.57 (± 0.07)/17.01	3.6/4.0	0.1	Gd
P-DARTS^19^	2.50/16.55	3.4/3.4	0.3	Gd
CDARTS^20^	2.48 (± 0.04)/15.69	3.9/3.9	0.3	Gd
GDAS^22^	2.93/18.38	3.4/3.4	0.2	Gd
Att-DARTS^25^	2.62 (± 0.10)/16.54	3.2/3.2	–	Gd
ASM-NAS^1^	2.59/15.60	3.1/3.1	0.6	Gd
FairDARTS-a^3^	2.54 (± 0.05)/-	2.8/-	0.4	Gd
DARTS+^4^	2.50 (± 0.11)/16.28	3.7/3.7	0.4	Gd
DARTS−^5^	2.59 (± 0.08)/17.51	3.5/3.3	0.4	Gd
PPCAtt-NAS	2.51 (± 0.08)/16.42	3.6/3.7	0.18	Gd

TE, EP, SC, SM, Mn, RL, Ev, SO, Gd stand for test error, evaluation stage parameters, the time consumed in the search stage, search method, manual search, Reinforcement Learning based NAS, Evolution based NAS, SMBO based NAS, and Gradient based NAS, respectively. In the second and third columns, the left side of the symbol ‘/’ is the result of CIFAR10 and the right side is the result of CIFAR100.

The comparison on the Fashion-MNIST is shown in Table 4. On Fashion-MNIST, we can see that the testing error of our PPCAtt-NAS method is 3.61% (± 0.05), and the time cost of the architecture search phase is 0.18 GPU days. All other methods have higher time cost than PPCAtt-NAS, and have a lower architecture accuracy. It can be seen that NAS methods are superior to manual-based methods. In summary, PPCAtt-NAS is advantageous over the other methods, especially in lower architecture search time cost and higher architecture accuracy.

**Table 4:** Comparison results on fashion-MNIST.

Architecture	TE (%)	EP (M)	SC (GPU-days)	SM
DenseNet^7^	4.61	25.6	–	Mn
ResNet^8^	5.10	11.1	–	Mn
NASNet-A^10^	3.66	2.5	1800	RL
AmoebaNet-A^12^	3.67	2.3	3150	Ev
PNAS^13^	3.89	2.5	225	SO
ENAS^2^	3.79	2.6	0.5	RL
DARTS^14^	3.77	3.4	1.6	Gd
ASM-NAS^1^	3.70	2.6	0.5	Gd
GDAS^22^	3.76	2.4	0.2	Gd
P-DARTS^19^	3.75	3.4	0.3	Gd
PPCAtt-NAS	3.61	3.6	0.18	Gd

To further validate the migratability of the architectures searched by this paper's method, we extend it to a larger dataset, the ImageNet dataset, for experimental validation, and the results are presented in Table 5. Table 5 shows that this paper's method outperforms most of the existing methods with an error rate of 24.6% (± 0.04). The accuracy of the PPCAtt-NAS method is slightly lower than that of the P-DARTS method, but our method reduces 40% of the architecture search time. In addition, we use data augmentation to further improve the architecture accuracy (PPCAtt-NAS-A in Table 5) and improve the accuracy by 0.3%, successfully outperforming all the methods with an error rate of 24.3% (± 0.02).

**Table 5:** Comparision results on ImageNet.

Architecture	Top-1 TE (%)	Top-5 TE (%)	EP (M)	Flops (M)	SC (GPU-days)	SM
Inception-v1^31^	30.2	10.1	6.6	1448	–	Mn
MobileNet^32^	29.4	10.5	4.2	569	–	Mn
NASNet-A^10^	26.0	8.4	5.3	564	2000	RL
NASNet-B^10^	27.2	8.7	5.3	488	2000	RL
NASNet-C^10^	27.5	9.0	4.9	558	2000	RL
AmoebaNet-A^12^	25.5	8.0	5.1	555	3150	Ev
AmoebaNet-B^12^	26.0	8.5	5.3	555	3150	Ev
AmoebaNet-C^12^	24.3	7.6	6.4	570	3150	Ev
NSGANet-A2^30^	25.5	8.0	4.1	466	27	Ev
PNAS^13^	25.8	8.1	5.1	588	225	SO
DARTS^14^	26.7	8.7	4.7	574	4	Gd
PC-DARTS^15^	25.1	7.8	5.3	586	0.1	Gd
P-DARTS^19^	24.4	7.4	4.9	557	0.3	Gd
Att-DARTS^25^	26.0	8.5	4.6	–	10	Gd
ASM-NAS^1^	25.4	8.1	5.5	–	0.55	Gd
GDAS^22^	26.0	8.5	5.3	581	1	Gd
FairDARTS-a^3^	26.3	8.3	3.6	417	0.4	Gd
EnTranNAS^21^	24.8	7.6	4.9	562	0.03	Gd
PPCAtt-NAS	24.6	7.6	5.1	619	0.18	Gd
PPCAtt-NAS-A	24.3	7.4	5.1	619	0.18	Gd

Top-1 TE, Top-5 TE, Flops stand for top-1 test error, top-5 test error, floating-point operations per second, respectively.

## Conclusion

In this paper, we propose a new progressive partially connected network architecture Search Strategy based on attention (PPCAtt-NAS). A progressive architecture search strategy is adopted to bridge the depth gap between two phases of the neural architecture search. Meanwhile, a progressive partial connection search scheme is implemented by gradually varying the channel sampling probability to reduce the computational cost of the architecture search phase. And an attention mechanism is utilized to improve the network architecture search performance, avoiding the performance collapse. Finally, extensive comparison experiments are carried on several publicly available datasets. The results show that our proposed search strategy achieves higher architecture performance compared to other state-of-the-art methods, and thus the effectiveness of our PPCAtt-NAS method has been verified.

## Data Availability

The datasets generated and/or analysed during the current study are available in the Pytorch repository, [https://pytorch.org/vision/stable/datasets.html].
